# Dural ectasia of the optic nerve sheath

**DOI:** 10.11604/pamj.2014.17.140.3755

**Published:** 2014-02-27

**Authors:** Hanane Hadj Kacem, Lehcen Hammani, Ali Ajana, Itimad Nassar

**Affiliations:** 1 Department of Radiology, Avicenne Hospital, Ministry of Health, Rabat, Morocco

**Keywords:** Optic nerve, sheath, dural ectasia, MRI

## Abstract

Optic nerve dural ectasia is a rare cause of optic nerve sheath enlargement due to the accumulation of CSF around the optic nerve with no associated pathology. It diagnosed by MRI studies and can follow benign or sometimes an unfavorable course. We describe the case of a 24-day-old female referred for a visual blurring, which we diagnosed as a dural ectasia of the optic nerve sheath by MRI and confirmed in surgical intervention. We present this case report to illustrate the classic imaging features of the disease.

## Introduction

Dural ectasia of the optic nerve is a rare cause of optic nerve sheath enlargement due to the accumulation of CSF around the optic nerve with no associated pathology [1]. The terms: optic hydrops, primary cyst of the optic nerve sheath, patulous subarachnoid space, cystic hygroma, arachnoid cyst, perioptic subtotal hygroma, and dural ectasia of the optic nerve sheath all have been used to describe this entity since its first description in 1918. The term optic nerve sheath meningocele was introduced by Garrity et al in 1990 [1, 2]. We prefer the term dural ectasia to describe the optic nerve sheath dilatation. We report the case of a 24-day-old female referred for a visual blurring, which we diagnosed as a dural ectasia of the optic nerve sheath by MRI and confirmed in surgical intervention.

## Patient and observation

A 24-year-old woman presented with a 8 month history of visual blurring with a slow decrease in visual acuity, and headache. Humphrey's visual field analysis revealed an enlarged blind spot. Dilated funduscopy showed an elevation of the left optic disc and choroidal folds above the nerve extending into the macula. General physical examination was normal.

**Figure 1:**
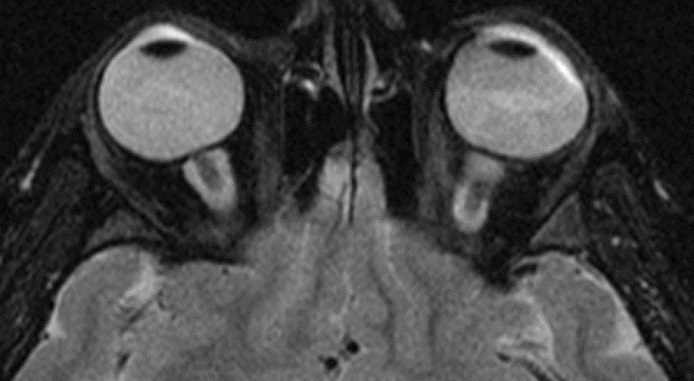
Orbital MRI; Axial view of T2-weighted MRI Hyperintense cerebrospinal fluid-intensity dilated sheath surrounding normal optic nerves (bull's eye)

**Figure 2:**
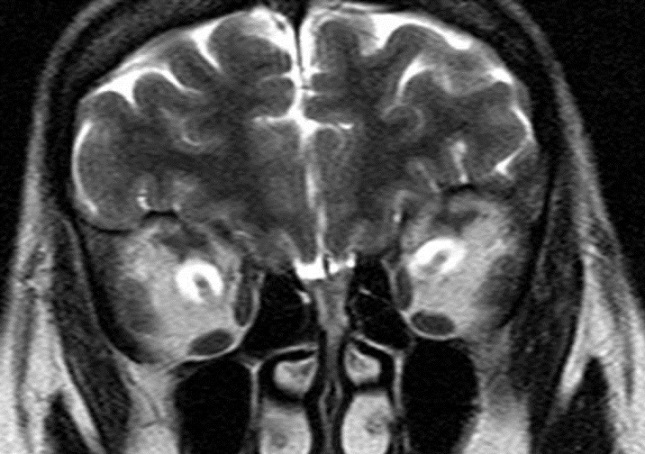
Orbital MRI; Coronal view of T2-weighted MRI Hyperintense cerebrospinal fluid-intensity dilated sheath surrounding normal optic nerves (bull's eye)

A magnetic resonance imaging (MRI) with fat-suppression and an off-axis, coronal and sagittal views were performed, a tubular-cystic enlargement of the optic nerve sheath was identified as containing a CSF (cerbrospinal fluid)-intensity lesion that was hyperintense on T2-weighted images (Figure 1, Figure 2). There was no evidence of tumor in the brain or orbit.

The subject was therefore, diagnosed as having dural ectasia of the optic nerve sheath, which is also known as optic nerve meningocoele, without intracranial hypertension. Once the diagnosis had been confirmed in surgical intervention. The patient remained stable over the next 4 months with the same visual acuity.

## Discussion

Dural ectasia of the optic nerve sheath (Optic neural sheath meningocele, optic nerve sheath cyst, arachnoid cyst, and perioptic hygroma, among others) is a saccular dilatation of the meninges surrounding the orbital portion of the optic nerve [2]. The lumen of the meningocele is filled with cerebrospinal fluid. It may occur primarily or secondarily in association with other orbital processes, such as meningioma, optic nerve pilocytic astrocytoma, and hemangioma [3]. More than 30 patients with dural ectasia of the optic nerve sheath have been described in the literature [3]. Thus, most common association with this disease appears to be NF1. There is little histopathologic material available on optic nerve ectasia. Other than the presence of a cyst, the meninges appear to be normal. The pathogenesis is mostly unknown [3, 4] Presenting symptoms are often related to involvement of the optic nerve, it present with changes in the visual acuity, visual field, proptosis, afferent papillary defect, enlarged blind spot, and optic nerve appearance [4].

The radiological examination of choice is MRI with high spatial resolution, fat-suppression with contrast techniques in off-axis, cronal and sagittal views [5]. Dural ectasia of optic nerve sheath appears as a prominent focal or segmental enlargement of the dural arachnoid sheath around the optic nerve; isointense with cerebrospinal fluid and a normal or thickened optic nerve (ʺbull's eyeʺ). Short TE/short TR spin echo sequences gives the best results [6]. This optic nerve dural ectasia may be associated with empty sella and enlarged subarachnoid cisterns, such as gasserian cisterns. MRI allows a more detailed differential diagnosis of optic nerve meningocele, including optic nerve tumors such as gliomas or meningiomass, especially in the cystic subtype of these tumors. Treatment of dural ectasia of the optic nerve sheath depends on optic nerve functions. The objective in traitement is to eliminate pressure on the optic nerve in order to prevent or reverse visual loss [7]. Corticosteroids are generally ineffective. If there is no optic nerve compression, observation is warranted. If there is compression, the best treatment appears to be surgical decompression of the optic nerve by removing the stretched dura mater and arachnoid over the cyst, allowing it to drain. Recurrence is possible if the cyst wall is not completely excised [8].

## Conclusion

Optic nerve dural ectasia is a saccular dilatation of the optic nerve sheath. It can be suspected based on the evidence of visual blurring or retrobulbar pressure. The radiological investigation of choice is MRI with techniques to emphasize high spatial resolution and optic nerve anatomy. Surgical intervention is reserved for severe cases of optic nerve decompression.
